# Promyelocytic Leukemia Zinc Finger Protein Activates GATA4 Transcription and Mediates Cardiac Hypertrophic Signaling from Angiotensin II Receptor 2

**DOI:** 10.1371/journal.pone.0035632

**Published:** 2012-04-27

**Authors:** Ning Wang, Gerald D. Frank, Ronghua Ding, Zhongjia Tan, Amita Rachakonda, Pier Paolo Pandolfi, Takaaki Senbonmatsu, Erwin J. Landon, Tadashi Inagami

**Affiliations:** 1 Department of Biochemistry, Vanderbilt University School of Medicine, Nashville, Tennessee, United States of America; 2 Center for Life Sciences, Beth Israel Deaconess Cancer Center, Boston, Massachusetts, United States of America; 3 Department of Pathology, Saitama Medical University, Saitama, Japan; Cardiovascular Research Institute Maastricht, - Maastricht University, The Netherlands

## Abstract

**Background:**

Pressure overload and prolonged angiotensin II (Ang II) infusion elicit cardiac hypertrophy in Ang II receptor 1 (AT_1_) null mouse, whereas Ang II receptor 2 (AT_2_) gene deletion abolishes the hypertrophic response. The roles and signals of the cardiac AT_2_ receptor still remain unsettled. Promyelocytic leukemia zinc finger protein (PLZF) was shown to bind to the AT_2_ receptor and transmit the hypertrophic signal. Using PLZF knockout mice we directed our studies on the function of PLZF concerning the cardiac specific transcription factor GATA4, and GATA4 targets.

**Methodology and Principal Findings:**

PLZF knockout and age-matched wild-type (WT) mice were treated with Ang II, infused at a rate of 4.2 ng·kg^−1^·min^−1^ for 3 weeks. Ang II elevated systolic blood pressure to comparable levels in PLZF knockout and WT mice (140 mmHg). WT mice developed prominent cardiac hypertrophy and fibrosis after Ang II infusion. In contrast, there was no obvious cardiac hypertrophy or fibrosis in PLZF knockout mice. An AT_2_ receptor blocker given to Ang II-infused wild type mice prevented hypertrophy, verifying the role of AT_2_ receptor for cardiac hypertrophy. Chromatin immunoprecipitation and electrophoretic mobility shift assay showed that PLZF bound to the GATA4 gene regulatory region. A Luciferase assay verified that PLZF up-regulated GATA4 gene expression and the absence of PLZF expression in vivo produced a corresponding repression of GATA4 protein.

**Conclusions:**

PLZF is an important AT_2_ receptor binding protein in mediating Ang II induced cardiac hypertrophy through an AT_2_ receptor-dependent signal pathway. The angiotensin II-AT_2_-PLZF-GATA4 signal may further augment Ang II induced pathological effects on cardiomyocytes.

## Introduction

Angiotensin (Ang) II is a potent vasoactive peptide, with strong effects on cardiac hypertrophy and congestive heart failure. Ang II has direct effects on elevated blood pressure, transactivation of the EGF receptor, and generation of reactive oxygen species [1]. Ang II binds to two major receptor subtypes, AT_1_ and AT_2_, with the most noted physiological and pathophysiological actions through the AT_1_ receptor. However, the AT_2_ receptor signaling and its significance have become more important, especially regarding cardiac remodeling mechanisms which are under-defined [2]. AT_2_ receptor expression is generally high in fetal tissues, declines rapidly postnatal to low levels in specific tissues, and then is re-expressed in certain pathological conditions such as cardiac hypertrophy, strongly suggesting important roles of the AT_2_ receptor in tissue growth and remodeling [3].

Mechanical stress alone induces cardiac hypertrophy in vivo [4]. Pressure overload elicits ventricular hypertrophy in AT_1a_ null mice [5], [6], [7], [8]. By contrast, in AT_2_ null mice with intact AT_1_ pressure overload or chronic Ang II infusion fails to elicit cardiac hypertrophy and interstitial fibrosis [9], [10]. Transplantation of wild type kidney to AT_1a_ null mice and subsequent Ang II infusion result in hypertension and cardiac hypertrophy indicating exclusive roles of the kidney in the etiology of hypertension [11] and a potential role of AT_2_ in cardiac hypertrophy. Also, the transfection of the AT_2_ receptor into cultured neonatal cardiomyocytes induces hypertrophy [12]. These results imply that in the heart Ang II activates AT_2_ to transmit a hypertrophic signal. This contrasts with other tissues where AT_2_ has been shown to elicit antigrowth and pro-apoptotic signals.

A widely accepted AT_2_ antihypertrophic signaling mechanism is a direct G-protein independent activation by AT_2_ of the protein tyrosine phosphatase SHP-1 that blocks growth factor signals [13], [14].

In search of the molecular mechanism which may provide material support for the unique cardiac hypertrophic response to the AT_2_ receptor action *in vivo*, we examined the hypothesis that a specific AT_2_-binding or modulating protein exists in the heart. The transcription factor promyelocytic leukemia zinc finger protein (PLZF) acts as a binding partner to the C-terminus of the AT_2_ receptor which was revealed by the yeast two-hybrid system and affinity binding technique. PLZF highly expressed in the heart activated gene transcription of the P85α regulatory subunit of phosphatidylinositol 3 kinase (p85α-PI3K). P85α further activated its downstream kinases Akt/PKB and p70^S6k^ resulting in cardiac hypertrophy [15].

PLZF is a transcription factor which contains 9 zinc fingers in the carboxyl terminal area and its amino terminus BTB/POZ domain mediates most biological functions of the zinc finger protein [16]. As an important transcription factor in cell differentiation and development, PLZF exhibits proapoptotic function in limb development [17] and an antiapoptotic role in developing testis [18]. PLZF regulates cyclin A2, c-myc, kit gene expression [19], [20], [21] and is involved in the signal of AT_2_ [15] and renin receptors [22].

The function of PLZF in different tissues and cells depends on its specific gene sequence context and different functional interaction partners [23], [24], [25].

Several cardiac transcription factors involved in fetal heart development have been identified including GATA4,5,6, NFAT 3, Nkx 2.5 and the transcription factor regulator HDACs [26], [27], [28], [29], [30], [31]. A reversion of these fetal gene expressions leads to maladaptive heart function [32]. Given that PLZF is an AT_2_ receptor binding protein in the heart; we hypothesized that it can interact with some of these transcription factors, specifically GATA4 in the heart. In the present study, we employed PLZF-/- mice to consolidate the hypothesis that the cardiac hypertrophic action of AT_2_ is regulated by PLZF in vivo.

## Methods

### Animals

**Figure 1:**
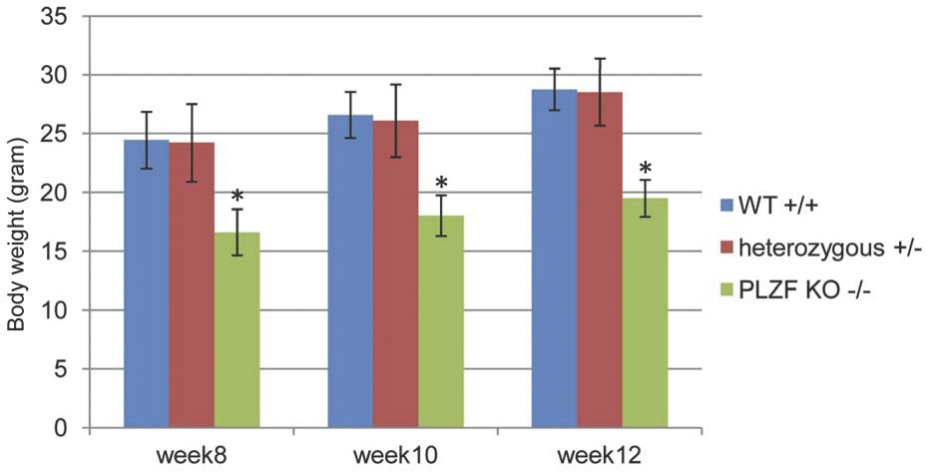
PLZF-/- mice weigh less than their wild type and heterozygote siblings. Mice were weighed for comparison. (* indicates p<0.05 vs all other groups; n = 12; data are mean±S.E.M).

All of the animal experimental protocols were approved by the Vanderbilt University Animal Use and Care Committee (A3227-01). *Agtr 2-/Y* mice were generated as described [33] and backcrossed to C57BL/6 genetic background. PLZF deficient mice were prepared by targeted disruption of the gene *Zfp145* in embryonic stem cells as described [18], they were backcrossed to C57BL/6 background. Because PLZF-/- animals are defective in hind limb bone formation, their access to chow was facilitated by providing food in a dish placed at the floor level. Ten to twelve weeks old PLZF-/- (n = 12) and WT (n = 10) male mice were used; their body weight is shown in Figure 1. In a pentobarbital-anesthetized (10 mg/kg I.P) mouse, an Ang II-impregnated pellet (Innovative Research of America) was placed under the shoulder skin. The pellets were prepared to release Ang II at a rate of 4.2 ng kg^−1^ min^−1^ for 21 or 60 days. For control, saline pellets were implanted. For the hydralazine group, wild type mice receiving Ang II pellets were given hydralazine 500 µg/ml in drinking water. For the AT_2_ receptor blockade group, wild type mice received a pellet which releases the AT_2_ blocker PD123319 at a rate of 15 µg kg^−1^ min^−1^ together with the Ang II pellet, as above.

### Blood Pressure Measurement

Mouse systolic blood pressure (SBP) was measured by the tail-cuff method and random samples (n = 6–7/group) verified the SBP by carotid arterial catheterization method. We trained mice for the manometry daily for 1 week prior to the experiment. Blood pressure was measured at 0, 7, 14, and 21 days during Ang II infusion.

### Echocardiography

Transthoracic echocardiography was performed using VisualSonics Vevo 770 High-Resolution Imaging System (VisualSonics Inc.) or Sonos 5500 (Agilent) for measurements of left ventricle (LV) internal diameter at end diastole and end systole, interventricular septal wall thickness (IVS) and posterior wall thickness (PW) as previously described [5].

### Histochemistry

Mouse hearts were isolated after perfusion with 40 mM KCl, and fixed in 4% par formaldehyde with PBS. Four µm paraffin sections were cut and stained with hematoxylin-eosin solution. The collagen fraction was calculated as ratio of the sum of the total area of interstitial fibrosis to the sum of the total connective tissue area plus the myocyte area in the entire visual field of a section. The area of perivascular fibrosis was determined as the ratio of the area of fibrosis surrounding the vessel wall to the total vessel area. Approximately 100 cells were examined in each heart.

### Western Blot

Mouse ventricles were ground on dry ice, or in liquid nitrogen in a pestle and mortar into a fine powder and homogenized in 1 mL of ice-cold TNF buffer (20 mM Tris-HCl (pH: 7.5), 150 mM NaCl, 2 mM EDTA, 1% NP-40, 50 mM NaF, 25 µg/ml aprotinin, 25 µg/ml leupeptin, 1 mM Na_3_VO_4_, 1 mM PMSF and 5 mM 2-mercaptoethanol). After 30 minutes exposure at 4°C, homogenates were centrifuged at 30,000×g for 30 minutes and supernatants were saved. Cardiac extracts were subjected to SDS-PAGE and transblotted onto PVDF membranes. Membranes were blocked, washed and incubated with primary and secondary antibodies. Protein bands were visualized by enhanced chemiluminescence (ECL) plus detection system.

### Competitive Reverse Transcription Polymerase Chain Reaction (RT-PCR)

Mouse ventricles were extracted in RNeasy Fibrous Tissue Mini kit (Qiagen). mRNA for AT1 and AT2 were determined by R-PCR, according to the published method [34].

### Elctrophoretic Mobility Shift Assay (EMSA)

Nuclear extracts (10 µg) from COS7 cells that were transfected with pCDNA4-PLZF plasmid was incubated with 2 nM ^32^p-labeled double-stranded oligonucleotide for 20 min at room temperature. Mouse anti-PLZF antibody (Santa Cruz Biotechnology, Santa Cruz, California) (2 µg) was added for the supershift assay. Protein-DNA complexes were electrophoresed in 4% native polyacrylamide gels and autoradioraphed. The sense strands of the oligonucleotides used in the EMSA were: 5′GGACAATCTAAAGTTCTTTCT-3′ (GATA4 native), and 5′GGACAATCATATGTTCTTTCT-3′ (GATA4 mutated), based on the GATA4 gene sequence 3 (NC_000008 NCBI).

### Chromatin Immunoprecipitation (ChIPs) Assay

ChIPs analysis was carried out with a commercial kit (Upstate Biotechnology) with some modifications to the manufacturer’s recommended conditions. Briefly, 2×10^6^ CHO-K1 cells, stably expressing AT_2_ and PLZF were cultured for 2 days and treated with 1% formaldehyde for protein-DNA cross-linking. The nuclear pellet was suspended in 600 µl of lysis buffer containing 1% SDS and 1× protease inhibitor cocktail, incubated for 10 min on ice, and sonicated sufficiently to shear the DNA to an average size of 500 to 1,000 bp. Chromatin was diluted 10-fold with ChIP dilution buffer, precleared with 80 µl of protein A-agarose and incubated with the anti-PLZF antibodies on a rotating shaker at 4°C overnight. After protein removal purified DNA was resuspended in 20 µl of H_2_O and analyzed by PCR. DNA for the input control was diluted 1∶100 before the PCR. Reactions were carried out in a volume of 50 µl, with initial denaturation at 94°C for 5 min, followed by 30 cycles of denaturation at 94°C for 30 sec, annealing at 54°C for 1 min, and extension at 72°C for 40 sec, followed by a 7-min terminal extension at 72°C.

### Luciferase Assay

Human *GATA4* gene 5′ upstream regulatory sequence was amplified by PCR with primers 5′-GGCATTGTACATTCTTCTCA-3′/5′-ACCTATTGGGGGCAGAAGC-3′, and was cut by KpnI and Bgl II. The promoter region of the *GATA4* gene was amplified with primers 5′GTAGCGCACGTCTCTTTCC-3′/5′GGTAGCACTTGGGCATTTTC-3′, and was cut by Bgl II and Hind III. These fragments were inserted to PGL3 basic vector plasmid (Promega). The dual luciferase assay system (Promega) was used to normalize for transfection efficiency by Renilla luciferase activity.

### Statistics

Data are expressed as mean±SEM. The significance of differences between control and experimental groups were evaluated using a one-way ANOVA with Student Newman-Keuls test. P<0.05 was considered statistically significant.

## Results

### Reduced Cardiac Hypertrophy and Fibrosis in Ang II-treated Homozygous PLZF-/- Mice

**Figure 2:**
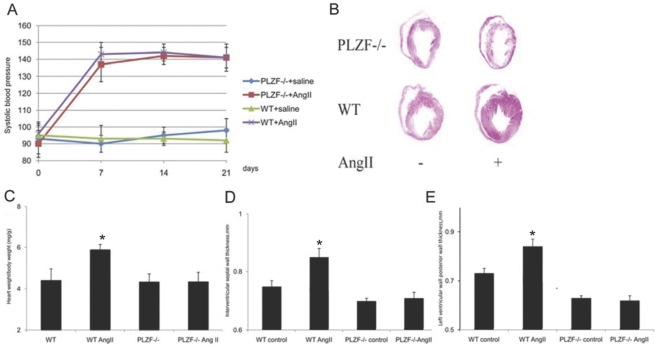
PLZF-/- mice show no obvious cardiac hypertrophy by Ang II infusion. (A) Systolic blood pressure increased in Ang II group (* indicates p<0.05 vs vehicle groups; n = 12; data are mean±S.E.M). (B) Mouse heart cross-section of the left ventricle. (C) The bar graph shows the ratio of heart weight (mg) to body weight (g) of mice from each group (* indicates p<0.05 vs all other groups; n = 8; data are mean±S.E.M.). (D)(E) Echocardiography data, (D) interventricular septal (IVS) and (E) left ventricular posterior wall (LVPW) thickness (* indicates p<0.05 vs all other groups; n = 8; data are mean±S.E.M.).

We reported that prolonged Ang II infusion [9] or pressure overload [10] failed to cause cardiac hypertrophy in AT_2_ deficient mice. We also reported that in cells and tissues high in PLZF expression the AT_2_ signal was transduced by PLZF [15]. We treated PLZF-/- and WT male mice with Ang II or with saline for 3 weeks by continuous infusion from subcutaneously embedded Ang II impregnated pellets to investigate the role of PLZF in Ang II induced cardiac hypertrophy. The systolic blood pressures of WT and PLZF-/- mice with Ang II infusion was raised to 140 mmHg (Figure 2A). The wild type mice showed a robust increase in the heart weight/body weight ratio whereas PLZF -/- mice showed little change in the ratio. (Figure 2 B,C).

**Figure 3:**
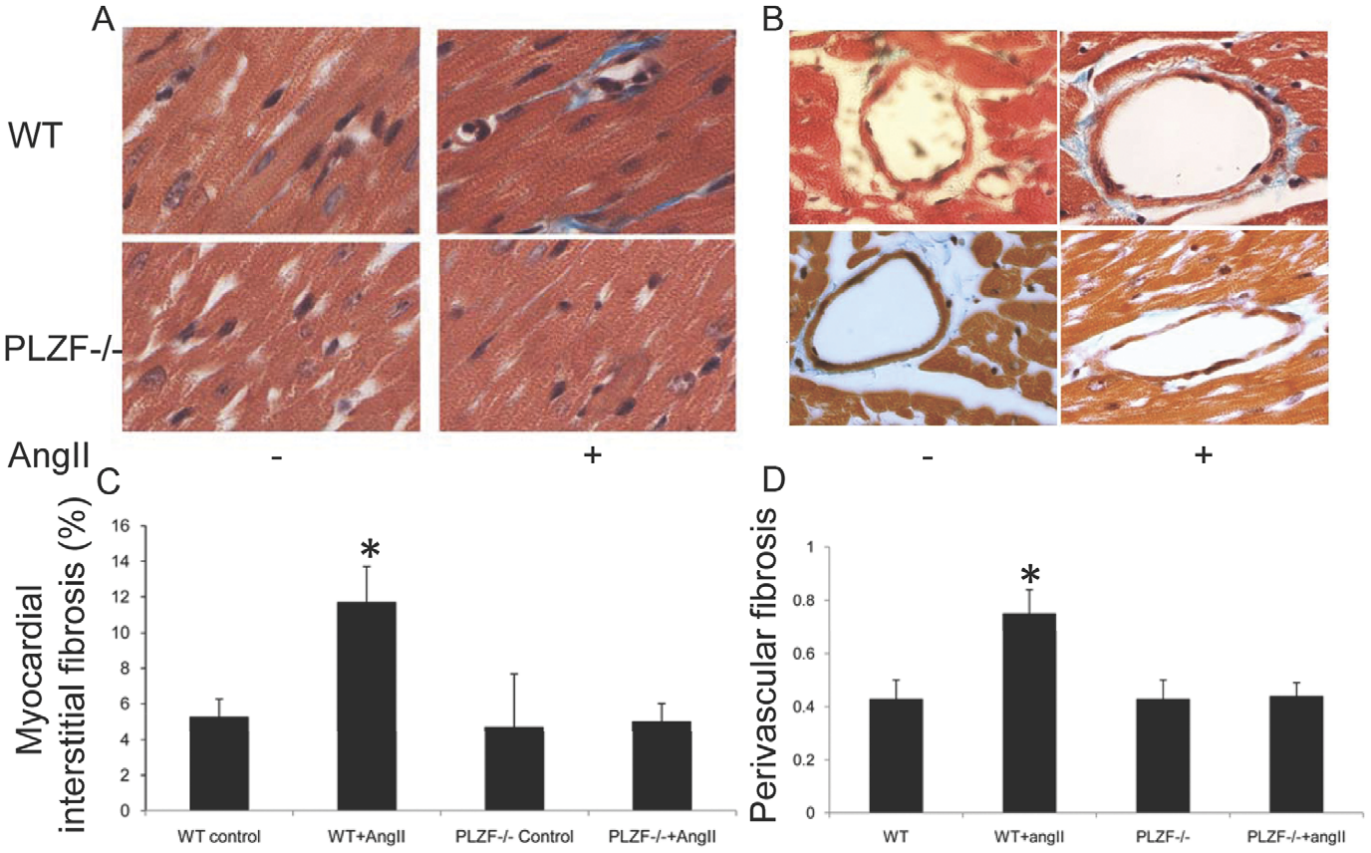
Less fibrosis is observed in Ang II infused PLZF-/- than wild type mice. Sections of left ventricle were stained with Masson’s trichrome to determine fibrosis. (A) Interstitial fibrosis. (* indicates significant changes at p<0.05 vs all other groups; n = 100; 5 mice for each group; data are mean±S.E.M.). (B) Perivascular fibrosis. (* indicates p<0.05 vs all other groups; n = 100; 5 mice for each group; data are mean±S.E.M.).

The Ang II treatment significantly increased wall thickness of IVS and PW in WT mice, but no obvious change was detected in PLZF-/- mice (Figure 2, D, E). Masson’s staining revealed that both interstitial and perivascular fibrosis (Figure 3,A,B) were significantly increased by Ang II infusion in WT mice, and no significant increase was seen in PLZF-/- mice after the Ang II infusion. Taken together, the reduced cardiac hypertrophy and fibrosis indicate that PLZF is one of the transcription factors mediating Ang II-induced cardiovascular remodeling.

### AT_2_ Receptor Pathway is Necessary for Ang II Induced Cardiac Hypertrophy

**Figure 4:**
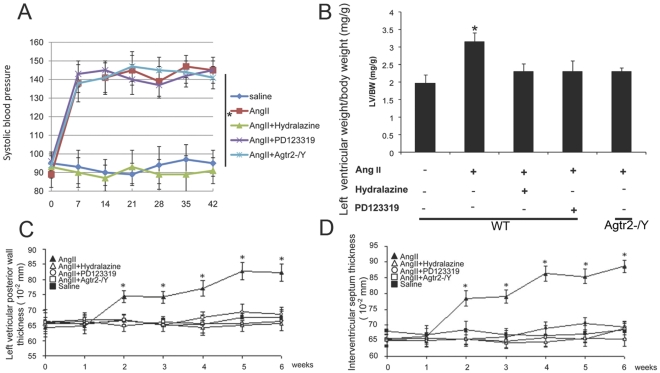
Elevated blood pressure and intact AT_2_ receptor are involved in eliciting Ang II-induced cardiac hypertrophy. (A) Systolic blood pressure increased in Ang II group, Ang II + PD123319 group and Ang II+ *Agtr2*-/Y group (*indicates p<0.05 versus vehicle; n = 15; data are mean±S.E.M.). (B) The bar graph shows the ratio of left ventricular weight (mg) to body weight (g) of mice from each group (* indicates p<0.05 vs all other groups; n = 7; data are mean±S.E.M.). (C)(D) Echocardiography data, line graphs show changes in IVS (C) and LVPW (D) in mice of each group (* indicates p<0.05 vs all other groups; n = 15; data are mean±S.E.M.).

We performed a series of *in vivo* studies, including wild type mice with control pellet, wild type mice with Ang II infusion, wild type mice with Ang II plus hydralazine, wild type mice with Ang II plus PD123319 and AT_2_ knockout mice with Ang II (Figure 4, A,B,C,D) to distinguish the functional roles of AT_2_ and pressure load in cardiac hypertrophy. We found that co-infusion of the specific AT_2_ blocker PD123319 with Ang II in wild type C57BL/6 mice suppressed Ang II-induced cardiac hypertrophy (Figure 4 B,C,D) confirming that AT_2_ is responsible for Ang II-stimulated hypertrophy. Ang II infusion into both wild type and AT_2_ knockout mice led to sustained elevation in systolic blood pressure (∼140 mmHg) which was normalized to 103–106 mmHg by a non-specific vasodilator hydralazine (Figure 4A). The hydralazine administration abolished Ang II induced cardiac hypertrophy (Figure 4 B,C,D). These results suggest that the elevated blood pressure is an important component of the increased hypertrophic response in AT_2_-dependent Ang II signals.

### AT_2_ Expression in Cardiac Ventricles is Promoted by Ang II Infusion in Wild Type Mice

Given evidence for the role of AT_2_ in ventricular hypertrophy in wild type mice receiving Ang II infusion (Fig. 4 B,C,D) we examined possible changes in expression levels of AT_1_ and AT_2_ during infusion of Ang II. *In vivo* cardiac AT_2_ receptor protein is expressed at about 20% of AT_1_ protein level in untreated C57BL6 mouse strain which is too low to be determined by competitive ligand binding assay [34]. By RT-PCR AT_2_ mRNA expression increased by 80% in 3 days and 180% in 7 days with±15% error, n = 4. Whereas AT_1_ mRNA did not increase in the first 3 days and showed a 100% increase in 7 days (with±19% error, n = 4). Almost 3-fold increase in AT_2_ mRNA suggests that Ang II will have a fairly strong effect on downstream events of the heart. These results are basically in agreement with observations reported by Fujii on double transgenic mouse expressing human renin and angiotensinogen which develops angiotensin dependent hypertension [34].

### PLZF Specifically Binds to the Upstream Flanking Region of *GATA4* Gene

**Figure 5:**
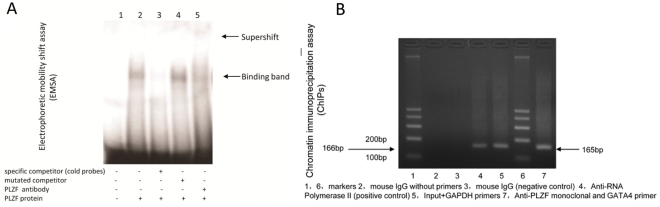
PLZF binds to the regulatory region of *GATA4* gene. (A) Elctrophoretic mobility shift assay (EMSA) verified the PLZF binding activity at *GATA4* gene. Lane 1: negative control with nuclear extract of COS7 cells not expressing PLZF. Lane 2: positive control with PLZF expressing cell nuclear extract. (B)Chromatin immunoprecipitation assay (ChIPs) analysis determined the PLZF occupancy in GATA4 gene at the chromatin level.

We previously reported that the AT_2_-PLZF-p85α-PI3K-Akt mechanism was involved in the AT_2_ mediated signaling pathway [15]. Considering that PLZF is an important transcription factor in development [17], [18] and cardiac hypertrophy [15], [22], we further studied the possibility of a transcription function of PLZF. According to Li et al. [36], a core consensus sequence for the specific binding of the PLZF is AT/GG/CTA/CA/CAGT. Based on MatInspector reports [37], a putative PLZF binding site is present in the human *GATA4* promoter region with a high core and matrix similarity (Sequence from NC_000008 NCBI). We performed an electrophoretic mobility shift assay (EMSA) to investigate whether it is a PLZF target. The results showed a positive band that was abolished by a competing mutant probe. Supershift assay with PLZF antibody showed a retarded band, which further confirmed the binding activity (Figure 5A). These results indicate the potential for PLZF to bind to the *GATA4* gene regulatory region. Further, chromatin immunoprecipitation assay (ChIPs) analysis verified that this *GATA4* gene sequence at the chromatin level was indeed occupied by PLZF. A CHO-K1-AT_2_-PLZF cell line with stable AT_2_ and PLZF expression was cultured for 2 days and treated with 1% formaldehyde to cross-link DNA and associated proteins. The chromatin was fragmented by sonication. The resulting PLZF-bound fragments were subjected to immunoprecipitation with PLZF antibody, and the chromatin-bound proteins were digested overnight with proteinase K in 200 mM NaCl at 65°C. DNA freed from the bound protein was identified by PCR which was performed with primers encompassing the putative PLZF binding sequence. As shown in Figure 5B, this GATA4 regulatory sequence was bound by PLZF.

### Upregulation of GATA4 Gene Expression through the AT_2_ and PLZF Signaling Pathway

**Figure 6:**
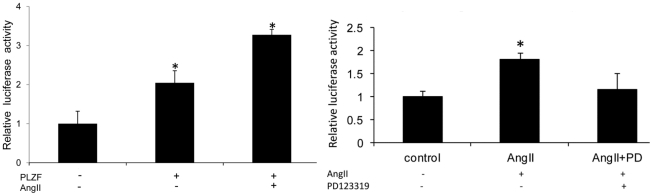
PLZF up-regulates GATA4 gene expression. (A) Luciferase reporter gene activity in PLZF co-transfected R3T3 cells (* indicates p<0.05 vs control group; n = 5; data are mean±S.E.M.). (B) Luciferase reporter gene activity in R3T3 cells which were pretreated with AT_2_ antagonist PD123319 for 1 hour before experiment. (* indicates p<0.05 vs all other groups; n = 5; data are mean±S.E.M.).

The present finding that the *GATA4* gene has a specific PLZF binding site in its upstream flanking region suggests that it may be a functional site that regulates transcription of the *GATA4* gene. Since we have found that Ang II-mediated cardiac hypertrophy depends on the expression of PLZF, and that PLZF activation is driven by AT_2_ in the heart [15]; and since GATA4 is one of the well known factors involved in cardiac development and hypertrophy [26], we examined the possible transcriptional role of PLZF on *GATA4* gene expression by luciferase assay in R3T3 cells which express the AT_2_ receptor but not AT_1_. The luciferase plasmid construction was based on pGL3-basal vector with a 904 bp insert from the upstream regulatory sequence of the human *GATA4* gene and a GATA4 promoter [35]. As shown in Figure 6A, PLZF-expressing R3T3 cells had a markedly elevated (200% of the basal level) luciferase transcription activity. When Ang II (0.1 mM) was added to the PLZF-transfected R3T3 cells, the luciferase activity was further elevated to 300% of the control level. These results support the hypothesis that PLZF up-regulates *GATA4* gene expression, and that the activation is further enhanced by Ang II action through the AT_2_ receptor.

Results in Figure 6B further confirm this conclusion as the non-peptide AT_2_ receptor antagonist PD123319 completely eliminated the enhanced effect of Ang II on luciferase activity.

### Elevated Expression of GATA4 Protein in Ang II-treated Wild Type Mouse Heart

**Figure 7:**
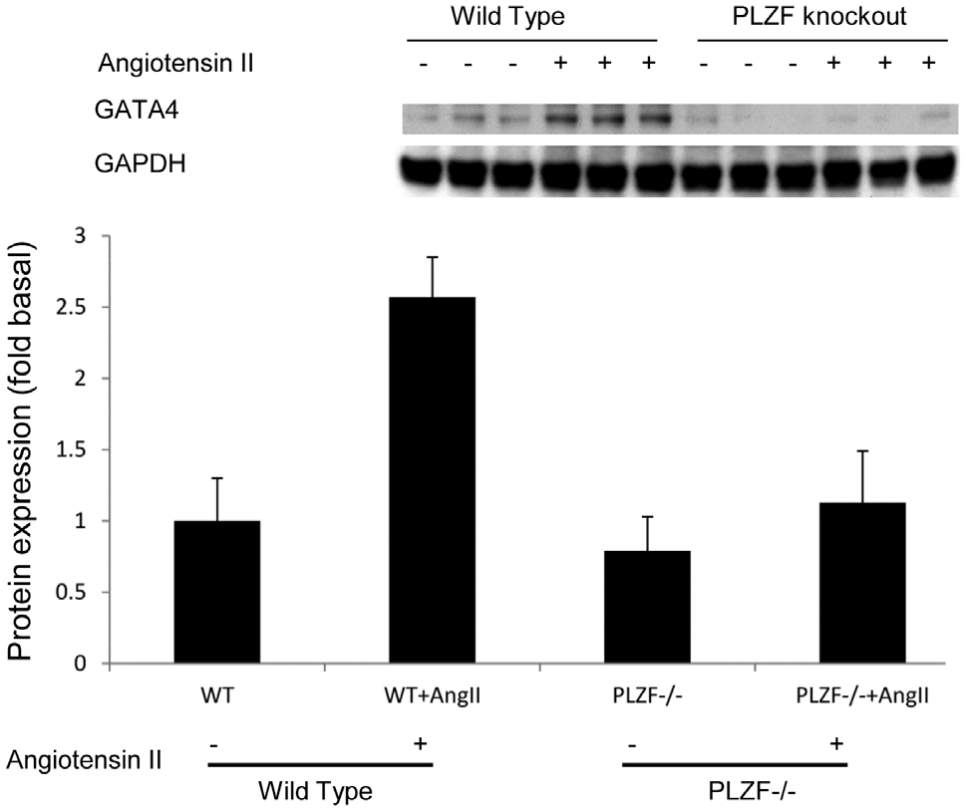
GATA4 protein expression is significantly elevated in the Ang II treated wild type mouse heart. Protein extracts from mice left ventricles were measured by Western blot (* indicates p<0.05 vs all other groups; n = 5; data are mean±S.E.M.). Ordinate axis indicates protein amount normalized by GAPDH, control is set to 1.0.

Given the ability of PLZF to increase GATA4 gene expression *in vitro*, we next examined whether the absence of PLZF expression *in vivo* produced a corresponding repression of GATA4 protein. PLZF-/- and wild type mice received Ang II infusion for 3 weeks. GATA4 protein was examined in the left ventricular extract by Western blotting using GATA4 polyclonal antibodies. GATA4 protein was markedly elevated after 3 weeks in WT mice, whereas in PLZF-/- no significant induction of GATA4 protein was recognized, as shown in Figure 7. This result provides further support to the conclusion that the *GATA4* gene is regulated by PLZF *in vivo*.

### Ang II Activates Cardiac GATA4 Signal to Target Genes

**Figure 8:**
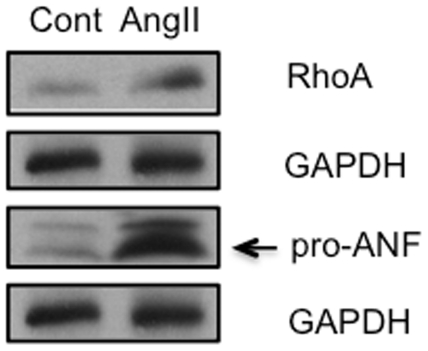
Ang II treatment of wild type mice for 3 weeks up regulates cardiac contents of RhoA and proANF, which are randomly selected targets of GATA4.

GATA4 controls numerous cardiac target genes. The question arose whether the upstream signal of the Ang II-AT2-PLZF signal generally reaches GATA4. To test such a possibility, we studied whether Ang II infusion for 3 weeks to wild type C57BL/6 mouse will stimulate synthesis of well known targets of GATA4 pro-atrial natriuretic factor (pro-ANF) [38] and RhoA [39]. Ang II infusion markedly increased ventricular pro ANF compared with controls (n = 3) by Western blot analysis of the target proteins (Figure 8). These results show Ang II signal, possibly via AT_2_, activates GATA4 target genes.

## Discussion

In the present study, we report evidence indicating PLZF, a Kruppel-like zinc finger protein highly expressed in the heart [40], is a crucial transcription factor that regulates cardiac hypertrophy through the AT_2_ receptor in response to Ang II. Ang II-activated AT_2_ receptor has been shown to bind PLZF and facilitate its nuclear translocation [15]. The present study shows that it up-regulates the expression of GATA4, a key cardiac morphogenic, hypertrophy and remodeling regulator [41].

PLZF was first recognized to fuse with retinoic acid receptor RARa in acute promyelocytic leukemia. PLZF suppresses gene transcription by recruiting corepressors to the gene regulation region and activate gene transcription with different molecular mechanism which has not been well defined [25]. PLZF is increasingly recognized as a key regulator in cell differentiation, growth or self-renewal process. Using PLZF knockout mice, we found suppressed cardiac hypertrophy (Fig. 2 B,C,D,E) and fibrosis (Fig. 3 A,B,C,D) in PLZF-/- mice subjected to chronic Ang II stimulation. Further experiments revealed PLZF regulated GATA4 expression, a vital factor in heart development and differentiation and remodeling [26], [41], [42]. GATA4 directly stimulates numerous cardiac-specific genes, including those of α- and β-myosin heavy chain genes which are key indices of cardiomyocyte hypertrophy [43], [44], [45]. Therefore, we believe that the expression of GATA4 directly regulated by PLZF is another important factor of the cardiac remodeling controlled by the transcription factor PLZF in the Ang II AT_2_ signaling pathway.

Senbonmatsu, et al previously showed that stimulation of the AT_2_ receptor [15] in cells expressing PLZF, such as cardiomyocytes, elicits binding of PLZF to the receptor and its subsequent translocation into the nucleus, where it up-regulates the p85α regulatory subunit of PI3K. The nuclear translocation was demonstrated by markedly increased PLZF in cardiocyte nuclei (by immunohistochemical staining) of Ang II-infused wild type mice and also in AT_2_-expressing R3T3 cells transfected with a PLZF-expression plasmid [15]. It is this AT_2_-mediated nuclear translocation of PLZF that accounts for the activation of p85α in the previous studies [15] and GATA4 in the present study. The activation of the PI3K/Akt signal leads to cellular hypertrophy due to stimulation of protein synthesis by p70^S6k^. Moreover, GSK3ß is another important downstream hypertrophic factor in the PI3K/Akt signal pathway. GSK3ß phosphorylates GATA4 to promote its export from the nucleus through the exportin, Crm1 [46]. Akt inhibits GSK3ß activity and enhances nuclear accumulation of GATA4. Taken together the Ang II-AT_2_ exerted multiple convergent effects directed to cardiac hypertrophy through activation of p85α PI3K and p70^S6k^, inhibition of GSK3ß to facilitate nuclear localization of GATA4 and stimulation of GATA4 transcription.

The role of the AT_2_ receptor in cardiac hypertrophy is not completely explained. The present findings that two of the arbitrarily selected GATA4 targets are activated by Ang II, presumably via AT_2_-PLZF system, seems to indicate that many of GATA4 targets may be activated by this mechanism. More detailed studies are necessary to generalize it as a new GATA activation mechanism. The AT_2_ initiated GATA4 activation and cardiac hypertrophy are novel and somewhat surprising in view of the generally recognized AT_2_ function directed to growth inhibition. However, since the present hypertrophic mechanism involves cardiac specific GATA4 and heart selectively (but not exclusively) expressed PLZF, it may be a uniquely heart specific response under *in vivo* condition. Both AT_1_ and AT_2_ are proportionally up-regulated in the hypertrophic heart [34], [47]. AT_1_ inhibition does not prevent left ventricular remodeling induced by pacing [48]. Pressure overload elicits ventricular hypertrophy in AT_1a_ null mice [5], [6], [7], [8]. By contrast, in AT_2_ null mice pressure overload or chronic Ang II infusion fails to elicit cardiac hypertrophy and interstitial fibrosis [9], [10]. Overexpression of AT_2_ receptor in transgenic mice induces left ventricular hypertrophy [49]. Ang II up-regulates the immediate early transcription factor ATF3 through AT_2_ signal pathway and induces left atrial hypertrophy [50]. Alternatively, deletion of either AT_1_ or AT_2_ markedly attenuates cardiac hypertrophy in natriuretic peptide receptor/guanylyl cyclase-A (GCA)-deficient mice [51]. Similar to these observations, our *in vivo* studies showed high blood pressure and AT_2_ were critical for Ang II induced cardiac hypertrophy. Abating pressure overload by hydralazine, blockade of AT_2_ receptor with the AT_2_ antagonist PD123319 or deletion of AT_2_ *(Agtr2-/Y)* prevents cardiac hypertrophy.

Cardiac AT_2_ is up-regulated by mechanical stretch [52] and pressure overload [34], [53]. Moreover, AT_2_ promotes ligand-independent, constitutive cardiomyocyte hypertrophy [54]. AT_1_ and AT_2_ receptors are considered to interact with each other to enhance the effects they mediate. Combined treatment with losartan and PD123319 proved to be more effective in attenuating the reflex increase in plasma adrenaline concentrations during insulin-induced hypoglycemia than either of the two Ang II receptor antagonists given alone [55]. Furthermore, the combination of both AT_1_ and AT_2_ receptor antagonists, at concentrations that each partly reduced inositol 1,4,5-trisphosphate (IP_3_), completely inhibited IP_3_ formation, suggesting that AT_1_ and AT_2_ cooperate in Ang II-mediated IP_3_ signal transduction for the actions of Ang II mediated by the IP_3_ signal transduction pathway [56].

Because GATA4 with AP-1 (activator of protein-1) up-regulates AT_1_ receptor expression [57], our study provides insight into a plausible mechanism of interaction between AT_1_ and AT_2_ signaling pathways, in which PLZF bridges AT_2_ and AT_1_ signaling through GATA4. Thus, AT_2_ could be an upstream cardiac hypertrophy factor of AT_1_ signaling. The interactions between AT_1_ and AT_2_ are complicated but the comprehensive understanding of these mechanisms could lead to better understanding of the therapeutic strategies of hypertension and cardiac hypertrophy. The present observation that AT_2_ receptor antagonist PD123319 inhibits the Ang II mediated cardiac hypertrophy is in agreement with our earlier observation with AT_2_ deficient mice which lost hypertrophic response to Ang II. It is important to note that normalization of blood pressure by hydralazine prevented cardiac hypertrophy (Fig. 2A) which indicates that pressure load is essential for the hypertrophy in agreement with existing reports [4], [34], [47], [57] as discussed above myocyte stretch and tension induce expression of AT_2_ [52], [53].

In summary, the present results of *in vivo* and *in vitro* studies demonstrate that PLZF activates GATA4 gene transcription and plays a significant role in the AT_2_-mediated cardiac hypertrophic response to Ang II.

## References

[1] de Gasparo M, Catt KJ, Inagami T, Wright JW, Unger T( 2000) International union of pharmacology. XXIII. The angiotensin II receptors. Pharmacol Rev..

[2] Porrello ER, Delbridge LM, Thomas WG (2009). The angiotensin II type 2 (AT_2_) receptor: an enigmatic seven transmembrane receptor. Front Biosci..

[3] Mehta PK, Griendling KK (2007). Angiotensin II cell signaling: physiological and pathological effects in the cardiovascular system. Am J Physiol Cell Physiol..

[4] Zou Y, Akazawa H, Qin Y, Sano M, Takano H (2004). Mechanical stress activates angiotensin II type 1 receptor without the involvement of angiotensin II. Nat Cell Biol..

[5] Harada K, Komuro I, Shiojima I, Hayashi D, Kudoh S (1998). Pressure overload induces cardiac hypertrophy in angiotensin II type 1A receptor knockout mice. Circulation..

[6] Harada K, Komuro I, Zou Y, Kudoh S, Kijima K (1998). Acute pressure overload could induce hypertrophic responses in the heart of angiotensin II type 1a knockout mice. Circ Res.. 82,.

[7] Hamawaki M, Coffman TM, Lashus A, Koide M, Zile MR (1998). Pressure-overload hypertrophy is unabated in mice devoid of AT_1a_ receptors. Am J Physiol..

[8] Kudoh S, Komuro I, Hiroi Y, Zou Y, Harada K (1998). Mechanical stretch induces hypertrophic responses in cardiac myocytes of angiotensin II type 1a receptor knockout mice. J Biol Chem..

[9] Ichihara S, Senbonmatsu T, Price EJ, Ichiki T, Gaffney FA (2001). Angiotensin II type 2 receptor is essential for left ventricular hypertrophy and cardiac fibrosis in chronic angiotensin II-induced hypertension. Circulation..

[10] Senbonmatsu T, Ichihara S, Price EJ, Gaffney FA, Inagami T (2000). Evidence for angiotensin II type 2 receptor-mediated cardiac myocyte enlargement during in vivo pressure overload. J Clin Invest..

[11] Crowley SD, Gurley SB, Herrera MJ, Ruiz P, Griffiths R (2006). Angiotensin II causes hypertension and cardiac hypertrophy through its receptors in the kidney. Proc Natl Acad Sci U S A..

[12] D’Amore A, Black MJ, Thomas WG (2005). The angiotensin II type 2 receptor causes constitutive growth of cardiomyocytes and does not antagonize angiotensin II type 1 receptor-mediated hypertrophy. Hypertension..

[13] Cui T, Nakagami H, Iwai M, Takeda Y, Shiuchi T (2001). Pivotal role of tyrosine phosphatase SHP-1 in AT_2_ receptor-mediated apoptosis in rat fetal vascular smooth muscle cell. Cardiovasc Res..

[14] Bedecs K, Elbaz N, Sutren M, Masson M, Susini C (1997). Angiotensin II type 2 receptors mediate inhibition of mitogen-activated protein kinase cascade and functional activation of SHP-1 tyrosine phosphatase.. Biochem J. 325 ( Pt.

[15] Senbonmatsu T, Saito T, Landon EJ, Watanabe O, Price EJ (2003). A novel angiotensin II type 2 receptor signaling pathway: possible role in cardiac hypertrophy. EMBO J..

[16] Collins T, Stone JR, Williams AJ (2001). All in the family: the BTB/POZ, KRAB, and SCAN domains. Mol Cell Biol..

[17] Barna M, Hawe N, Niswander L, Pandolfi PP (2000). Plzf regulates limb and axial skeletal patterning. Nat Genet..

[18] Costoya JA, Hobbs RM, Barna M, Cattoretti G, Manova K (2004). Essential role of Plzf in maintenance of spermatogonial stem cells. Nat Genet..

[19] Yeyati PL, Shaknovich R, Boterashvili S, Li J, Ball HJ (1999). Leukemia translocation protein PLZF inhibits cell growth and expression of cyclin A. Oncogene..

[20] McConnell MJ, Chevallier N, Berkofsky-Fessler W, Giltnane JM, Malani RB, et al (2003). Growth suppression by acute promyelocytic leukemia-associated protein PLZF is mediated by repression of c-myc expression. Mol Cell Biol..

[21] Filipponi D, Hobbs RM, Ottolenghi S, Rossi P, Jannini EA (2007). Repression of kit expression by Plzf in germ cells. Mol Cell Biol..

[22] Schefe JH, Menk M, Reinemund J, Effertz K, Hobbs RM (2006). A novel signal transduction cascade involving direct physical interaction of the renin/prorenin receptor with the transcription factor promyelocytic zinc finger protein. Circ Res..

[23] Ozato K (2009). PLZF outreach: a finger in interferon’s pie. Immunity..

[24] Xu D, Holko M, Sadler AJ, Scott B, Higashiyama S (2009). Promyelocytic leukemia zinc finger protein regulates interferon-mediated innate immunity. Immunity..

[25] Kolesnichenko M, Vogt PK (2011). Understanding PLZF. Cell Cycle..

[26] Molkentin JD (2000). The zinc finger-containing transcription factors GATA-4,-5, and -6. Ubiquitously expressed regulators of tissue-specific gene expression. J Biol Chem..

[27] Durocher D, Chen CY, Ardati A, Schwartz RJ, Nemer M (1996). The atrial natriuretic factor promoter is a downstream target for Nkx-2.5 in the myocardium. Mol Cell Biol..

[28] Tu VC, Sun H, Bowden GT, Chen QM (2007). Involvement of oxidants and AP-1 in angiotensin II-activated NFAT3 transcription factor. Am J Physiol Cell Physiol..

[29] Trivedi CM, Luo Y, Yin Z, Zhang M, Zhu W (2007). Hdac2 regulates the cardiac hypertrophic response by modulating Gsk3 beta activity. Nat Med..

[30] Ago T, Liu T, Zhai P, Chen W, Li H (2008). A redox-dependent pathway for regulating class II HDACs and cardiac hypertrophy. Cell..

[31] Olson EN (2006). Gene regulatory networks in the evolution and development of the heart. Science..

[32] Olson EN, Schneider MD (2003). Sizing up the heart: development redux in disease. Genes Dev..

[33] Ichiki T, Labosky PA, Shiota C, Okuyama S, Imagawa Y (1995). Effects on blood pressure and exploratory behaviour of mice lacking angiotensin II type-2 receptor. Nature..

[34] Fujii N, Tanaka M, Ohnishi J, Yukawa K, Takimoto E (1995). Alteration of angiotensin II receptor contents in hypertrophied hearts. Biochem Biophys Res Commun..

[35] Ohara Y, Atarashi T, Ishibashi T, Ohashi-Kobayashi A, Maeda M (2006). GATA-4 gene organization and analysis of its promoter. Biol Pharm Bull..

[36] Li JY, English MA, Ball HJ, Yeyati PL, Waxman S (1997). Sequence-specific DNA binding and transcriptional regulation by the promyelocytic leukemia zinc finger protein. J Biol Chem..

[37] Cartharius K, Frech K, Grote K, Klocke B, Haltmeier M (2005). MatInspector and beyond: promoter analysis based on transcription factor binding sites. Bioinformatics..

[38] Akazawa H, Komuro I (2003). Roles of cardiac transcription factors in cardiac hypertrophy. Circ Res..

[39] Charron F, Tsimiklis G, Ancand M, Robitaille L, Liang Q (2001). Tissue-specific GATA factors are transcriptional effectors of the small GTPase RhoA. Genes Dev..

[40] Cook M, Gould A, Brand N, Davies J, Strutt P (1995). Expression of the zinc-finger gene PLZF at rhombomere boundaries in the vertebrate hindbrain. Proc Natl Acad Sci U S A..

[41] Liang Q, Molkentin JD (2002). Divergent signaling pathways converge on GATA4 to regulate cardiac hypertrophic gene expression. J Mol Cell Cardiol..

[42] Oka T, Maillet M, Watt AJ, Schwartz RJ, Aronow BJ (2006). Cardiac-specific deletion of GATA4 reveals its requirement for hypertrophy, compensation, and myocyte viability. Circ Res..

[43] Adkins NL, Hagerman TA, Georgel P (2006). GAGA protein: a multi-faceted transcription factor. Biochem Cell Biol..

[44] Durocher D, Nemer M (1998). Combinatorial interactions regulating cardiac transcription. Dev Genet..

[45] Temsah R, Nemer M (2005). GATA factors and transcriptional regulation of cardiac natriuretic peptide genes. Regul Pept..

[46] Morisco C, Seta K, Hardt SE, Lee Y, Vatner SF (2001). Glycogen synthase kinase 3beta regulates GATA4 in cardiac myocytes. J Biol Chem..

[47] Matsubara H (1998). Pathophysiological Role of angiotensin II type 2 receptor in cardiovascular and renal diseases. Circ Res..

[48] Clair MJ, Krombach RS, Coker ML, Heslin TL, Kribbs SB (1998). Angiotensin AT_1_ receptor inhibition in pacing induced heart failure: effects on left ventricular myocardial collagen content and composition. J Mol Cell Cardiol..

[49] Yan X, Price RL, Nakayama M, Ito K, Schuldt AJ (2003). Ventricular-specific expression of angiotensin II type 2 receptors causes dilated cardiomyopathy and heart failure in transgenic mice. Am J Physiol Heart Circ Physiol..

[50] Hasin T, Elhanani O, Abassi Z, Hai T, Aronheim A (2011). Angiotensin II signaling up-regulates the immediate early transcription factor ATF3 in the left but not the right atrium. Basic Res Cardiol..

[51] Li Y, Saito Y, Kuwahara K, Rong X, Kishimoto I (2009). Guanylyl cyclase-A inhibits angiotensin II type 2 receptor-mediated pro-hypertrophic signaling in the heart..

[52] Kijima K, Matsubara H, Murasawa S, Maruyama K, Mori Y (1996). Mechanical stretch induces enhanced expression of angiotensin II receptor subtypes in neonatal rat cardiac myocytes.. Circ.

[53] Yayama K, Horii M, Hiyoshi H Takano M, Okamoto H (2004). Up-regulation of angiotensin II type 2 receptor in rat thoracic aorta by pressure overload. J Pharmacol Exp Ther..

[54] D’Amore A, Black J, Thomas W (2005). The AT_2_ receptor causes constitutive growth of cardiomyocytes and does not antagonize AT_1_ receptor-mediated hypertrophy. Hypertension..

[55] Worck RH, Frandsen E, Ibsen H, Petersen JS (1998). AT_1_ and AT_2_ receptor blockade and epinephrine release during insulin-induced hypoglycemia. Hypertension..

[56] Goutsouliak V, Rabkin SW (1998). Comparison of angiotensin II type-1 and type-2 receptor antagonists on angiotensin II-induced IP3 generation in cardiomyocytes. Gen pharmacol..

[57] Herzig TC, Jobe SM, Aoki H, Molkentin JD, Cowley AWJ (1997). Angiotensin II type 1a receptor gene expression in the heart: AP-1 and GATA-4 participate in the response to pressure overload. Proc Natl Acad Sci U S A..

